# Adoption of Secure Pork Supply Plan Biosecurity by U.S. Swine Producers

**DOI:** 10.3389/fvets.2019.00146

**Published:** 2019-05-28

**Authors:** Christopher C. Pudenz, Lee L. Schulz, Glynn T. Tonsor

**Affiliations:** ^1^Department of Economics, Iowa State University, Ames, IA, United States; ^2^Department of Agricultural Economics, Kansas State University, Manhattan, KS, United States

**Keywords:** animal health economics, biosecurity adoption, foreign animal diseases, Secure Pork Supply Plan, pig, swine

## Abstract

There is mounting concern about the negative animal health and supply chain consequences of animal disease outbreaks in the United States. Recent disease outbreaks have drawn attention to the need for additional understanding of biosecurity efforts to reduce disease frequency, spread, and impact. Biosecurity is a key component of the Secure Pork Supply (SPS) Plan designed to provide business continuity in the event of a foreign animal disease outbreak as well as help protect operations from endemic diseases. Core biosecurity recommendations outlined in the SPS Plan are a written site-specific biosecurity plan and implementation of a perimeter buffer area and a line of separation. To-date, no benchmarking of SPS Plan biosecurity implementation has been done. Utilizing data from a 2017 survey of U.S. swine producers, this study shows that SPS Plan biosecurity adoption varies and is affected by how feasible producers believe implementation of each biosecurity practice is on their operation. Furthermore, binomial logit regression analyses indicate producer and operation demographics and producer risk attitudes and perceptions affect biosecurity adoption. Conditional probabilities reveal that adoption of biosecurity practices is overwhelmingly complementary, suggesting that one biosecurity practice likely increases marginal efficacy of another biosecurity practice. The insights this study provides regarding the complexities of biosecurity adoption are vitally important to both educators and policy makers.

## Introduction

African swine fever (ASF), classical swine fever (CSF), and foot and mouth disease (FMD) are highly contagious transboundary animal diseases. An outbreak of one of these diseases in a country poses a severe threat to animal health and animal agriculture and would have significant economic consequences. Because of their potential for serious and rapid spread irrespective of borders and their ability to cause serious economic consequences and impact trade of animals and animal products, these foreign animal diseases (FADs) are reportable to and monitored by the World Organization for Animal Health (OIE). The United States has maintained ASF-, CSF-, and FMD-free status, but with these diseases present in many other countries, including the unsettling recent global ASF developments, the risk of introduction and spread is at the height of U.S. fears, considerations, and planning.

If ASF, CSF, or FMD were confirmed in the United States, response strategies for controlling and stopping the spread of these animal diseases would likely be far-reaching. It is reasonably certain that as a result of an outbreak of one of these diseases all movement of animals from affected industries would come to a complete halt, as would U.S. meat exports. Depending on the severity of the outbreak and the method used to contain the disease, some markets could remain closed for an extended period of time.

The Secure Pork Supply (SPS) Plan provides opportunities for pork producers through premise identification, enhanced biosecurity implementation, surveillance and sample collection, and movement monitoring to voluntarily prepare before a FAD outbreak. Having the SPS Plan implemented prior to an FAD outbreak is intended to enhance coordination and communication between all stakeholders, speed up a successful FAD response, and eventually enable the issuance of animal movement permits after the extent of the outbreak is understood. Collectively, this should help support continuity of business for participating producers and allied industries^1^.

In order for the SPS Plan to effectively meet the goals it has set forth, a minimum level of participation is necessary, and the full benefits of the plan are likely only realized with a high level of participation. To date, no benchmarking of SPS Plan implementation has been done. This highlights a critical need that we aim to meet in this study by identifying and explaining producer implementation of SPS Plan components, namely enhanced biosecurity adoption. Understanding adoption, or lack thereof, is important for improving program targeting and policy deliberations as well as for increasing voluntary participation.

## Background and Work Needed

The United States is a significant producer and consumer of pork and pork products, and any event that would interrupt exports, imports, or movement of animals within the country would have serious economic consequences. In 2017, the United States was the world's second-largest exporter of pork and pork products, with exports averaging 22% of domestic commercial pork production (1). Live imports into the United States are important to the domestic swine industry, with imports of all hogs and pigs into the United States during 2017 totaling 5.6 million head (2). Specifically, feeder pig imports from Canada during 2017 accounted for 4.8 million head (2). Internally, U.S. pork production depends on the extensive movement of animals. Of the approximately 171.4 million hogs and pigs marketed in the United States in 2017, 55.2 million were shipped across state lines for feeding or breeding purposes (3). With this being the case, any factor that might restrict exports, imports, and state-to-state shipments would have serious economic implications for producers and the broader economy. Therefore, it is important to identify, prior to an outbreak, potential procedures and plans that may mitigate the consequences and maintain continuity of business by reestablishing movements and trade as quickly as possible.

During a FAD outbreak, as is the case with any other disease, it is a producer's responsibility to keep his/her animals from becoming infected. As such, while the responsibility for preventing the introduction of a FAD into the United States is primarily assigned to the U.S. Department of Agriculture Animal and Plant Health Inspection Service (USDA-APHIS) and other government agencies, producers are the first line of defense in preparedness and are critical to response and recovery efforts. Work on the SPS Plan by federal and state officials, industry, and academia has created recommendations for enhanced biosecurity practices that are designed to prevent the introduction and spread of disease agents onto or off of a production site. The specific practices are crafted based on knowledge about FMD, CSF, and ASF, but they also help protect production sites from endemic diseases (4).

As described by Levis and Baker (5), biosecurity is comprised of bio-exclusion, bio-management, and bio-containment. Bio-exclusion aims to prevent the introduction of a disease into a herd or system, bio-management seeks to minimize the impact of diseases that have already been introduced into a herd or system, and bio-containment strives to prevent the spread of diseases from one herd or system to another, thereby protecting the rest of the supply chain (5). Even though bio-containment would be the most vital of the three components in the event of a FAD outbreak in the United States, this component often receives the least amount of attention from producers (5). The SPS Plan outlines enhanced biosecurity measures that, in addition to reflecting bio-exclusion and bio-management, contribute directly to bio-containment. Adoption of these recommended biosecurity practices would be one component in positioning operations (premises) with animals that have no evidence of infection during the outbreak to move animals to processing or another pork production premises under a movement permit and maintain domestic markets.

The SPS Plan could also help maintain continuity of business because it could be instrumental in compartmentalization and regionalization efforts. According to FAO and OIE (6), compartmentalization and regionalization (also known as zoning) are two disease management strategies that seek, through use of preventative biosecurity practices and separation of animal populations, to distinguish animal populations with differentiable health status. Whereas compartmentalization deals primarily with management and biosecurity within the establishments comprising the compartment, zoning focuses more on natural or human-made barriers and other geographic features (6). The disease-free status of these compartments and zones could promote continuity of business and prevent interruptions to, or reestablish, international trade (7). Compartmentalization has not been fully implemented by the United States for any disease agent to date and will depend on the recognition of the status of these compartments by international trading partners (6), but zoning helped maintain safe trade in poultry and poultry products during the highly pathogenic avian influenza outbreak in the United States in 2015 (8). The enhanced biosecurity measures recommended in the SPS biosecurity guidelines could contribute to compartmentalization (in particular) and zoning as they could aid producers in providing assurances to pertinent officials that they are not contributing to the spread of disease nor putting their own animals at risk of exposure.

An operation's ability to adopt, implement, and sustain a biosecurity intervention or process such as the SPS Plan is complex. As highlighted by Levis and Baker (5), the use of biosecurity measures differs widely among operations for a wide variety of reasons which include type of swine operation, geographic location, and epidemiological situation, which refers to causes, distribution, and control of diseases in the herd. Moore et al. (9) suggest many potential causes for producers deciding not to implement biosecurity recommendations, among which are an unawareness about the potential risks both to their operation and the entire industry, a miscalculation of costs compared to benefits, and confusion regarding which recommendations to adopt. Complicating the situation even further is, as Hennessy [(10), p. 70] notes, “Prevention involves making resource allocation choices about low probability risks that may materialize in the indefinite future. People are not particularly good at making such decisions, tending to overemphasize some risks and place too much weight on the recent past.”

Most data concerning producer decision making regarding biosecurity adoption is often incomplete or lacks the requisite depth for rigorous analysis. Lists of recommended biosecurity practices have been created by various entities (9), but there has been little research on adoption of these recommended biosecurity practices by swine producers in the United States. This makes intuitive sense given that, until the porcine epidemic diarrhea virus (PEDV) outbreak in 2013-14, most pork producers in the United States had not personally experienced a large emerging animal disease outbreak on their operations during their lifetimes, so adoption of biosecurity in U.S. pork production historically has been primarily precautionary and voluntary. This biosecurity paradigm is still generally in place today, but the PEDV outbreak did heighten awareness of new and improved biosecurity that proved beneficial against PEDV and a host of other pathogens and likely led to implementation of more complete and stringently suggested biosecurity plans. Still, existing data are mainly descriptive and lack the depth to fully understand producer decision making.

The literature on biosecurity adoption by swine producers in other countries is more comprehensive likely due to the number and type of animal disease outbreaks and damages incurred as well as both the existence of permit/assurance bonding schemes where it is the owners' responsibility to keep their animals free of disease and livestock disease insurance products available that offer reduced premiums for owners practicing good biosecurity (11). In particular, many studies have addressed livestock biosecurity adoptions in Europe, with recent examples including Simon-Grifé et al. (12) in Spain, Sahlström et al. (13) in Finland, and Postma et al. (14) in Belgium, France, Germany, and Sweden. Some of these studies identify producer and operation characteristics that influence adoption, but there has been little research on such impacts in the United States. Because SPS Plan enhanced biosecurity implementation is precautionary and voluntary, producer perceptions and characteristics of their operations are certainly important drivers of adoption.

The goal of this analysis is to first identify producer views on the feasibility of implementation of SPS Plan enhanced biosecurity recommendations on their operation. Of interest is whether feasibility (i.e., practicality of affordable implementation) may help explain lower-than-expected adoption of recommended biosecurity measures. Second, this analysis seeks to determine what type of producers (and operations) have implemented the SPS Plan enhanced biosecurity guidelines. Knowledge of these characteristics will help program administrators and educators better serve current participants as well as identify the characteristics of producers not currently participating and thus enable more efficient resource allocation in efforts to expand participation. Furthermore, since a biosecurity program is only as good as its weakest point, there is a need to understand what specific practices may increase adoption of other practices. Therefore, we also examine the complementary nature of biosecurity adoption. Altogether, this study provides the first comprehensive analysis of producer participation in the SPS Plan and should be critically valuable in future management of the SPS Plan and related programs.

## Materials and Methods

### Survey Instrument and Data

The survey procedures were approved by the Iowa State University Office for Responsible Research, Institutional Review Board. All methods for data collection and gathering were performed in accordance with the relevant regulations and in compliance with the received guidelines. Completion of the questionnaire by survey participants constituted implied consent.

A questionnaire developed by researchers and extension professionals and administered by the Iowa State University Center for Survey Statistics and Methodology was used in collecting information from swine producers in Iowa, Illinois, Indiana, Kansas, Minnesota, Michigan, Missouri, Nebraska, North Carolina, Ohio, Oklahoma, South Dakota, and Wisconsin. The 13 states surveyed represent 50% of U.S. hog operations and 91% of the U.S. hog inventory (15).

The survey questions were programmed for online application using Qualtrics survey software (Qualtrics, Provo, UT). The sampling frame used in selecting producers to survey was developed from state pork producer association membership lists. Survey data was collected using the online survey mechanism from March 23 through June 1, 2017. Information collected in the survey included producer and operation demographic characteristics, responses to how feasible implementation of SPS Plan biosecurity practices are on a producer's operation, and data detailing producers' use of SPS Plan biosecurity practices. Completed or partially completed surveys from 371 producers were received. Table 1 reports selected characteristics of the survey respondents. Further details regarding the survey instrument and sample design, data collection procedures, and a comprehensive summary of the data are available in Pudenz et al. (16).

**Table 1:** Characteristics of survey respondents.

**Variable**	**Description**	***N***	**Mean**	**Std. dev**.
AGE	Age of producer (in years)	276	53.366	12.107
COLLEGE	= 1 if 4 year college degree or graduate degree; 0 otherwise	279	0.563	0.497
**WHAT OPERATION TYPE BEST DESCRIBES YOUR HOG OPERATION?**				
FARROWFINISH	= 1 if farrow to finish; 0 otherwise	371	0.299	0.459
BREEDING	= 1 if breeding/farrowing or nursery; 0 otherwise	371	0.105	0.307
WEANFINISH	= 1 if wean to finish; 0 otherwise	371	0.364	0.482
FINISH	= 1 if finish; 0 otherwise	371	0.173	0.378
OTHEROPERATION	= 1 if boar stud or gilt developer unit or other operation type; 0 otherwise	371	0.059	0.237
**WHICH BUSINESS ARRANGEMENT BEST DESCRIBES THE AGREEMENT UNDER WHICH YOU ARE PRESENTLY PRODUCING HOGS?**				
INDEPENDENT	= 1 if independent producer; 0 otherwise	369	0.485	0.500
INTEGRATOR	= 1 if contractor or integrator; 0 otherwise	369	0.106	0.308
CONTRACTGROWER	= 1 if contract grower (contractee); 0 otherwise	369	0.360	0.481
OTHERBUSINESS	= 1 if other business arrangement; 0 otherwise	369	0.049	0.216
IOWA	= 1 if Iowa pork producer; 0 otherwise	371	0.604	0.490
PRODUCTIONSITES	Number of separate productions sites (unique premise ID, unique address) in 2016	351	16.436	57.481
HIGHRATING	= 1 if producer's operation biosecurity is perceived to be higher than other operations in the area; 0 otherwise	336	0.830	0.376
REPORTABLE	= 1 if a producer's operation has experienced PRRSV and/or PEDV in the past 3 years; 0 otherwise	354	0.684	0.466
**HOW MANY TIMES IN THE NEXT 100 YEARS DO YOU THINK A TIER 1 DISEASE OUTBREAK WILL OCCUR IN THE U.S. SWINE INDUSTRY?**				
NOOUTBREAKS	= 1 if no outbreaks expected; 0 otherwise	298	0.091	0.288
ONEOUTBREAK	= 1 if one outbreak expected; 0 otherwise	298	0.235	0.425
TWOOUTBREAKS	= 1 if two or more outbreaks expected; 0 otherwise	298	0.674	0.469

### Feasibility and Implementation Cross Tabulation Analysis

The SPS Plan emphasizes biosecurity concepts that all pork production sites must implement to help protect their animals from endemic diseases and to be prepared in the event of an FAD outbreak in the United States. These include a written site-specific plan, perimeter buffer area (PBA), and line of separation (LOS) ^2^,^3^.

**Figure 1:**
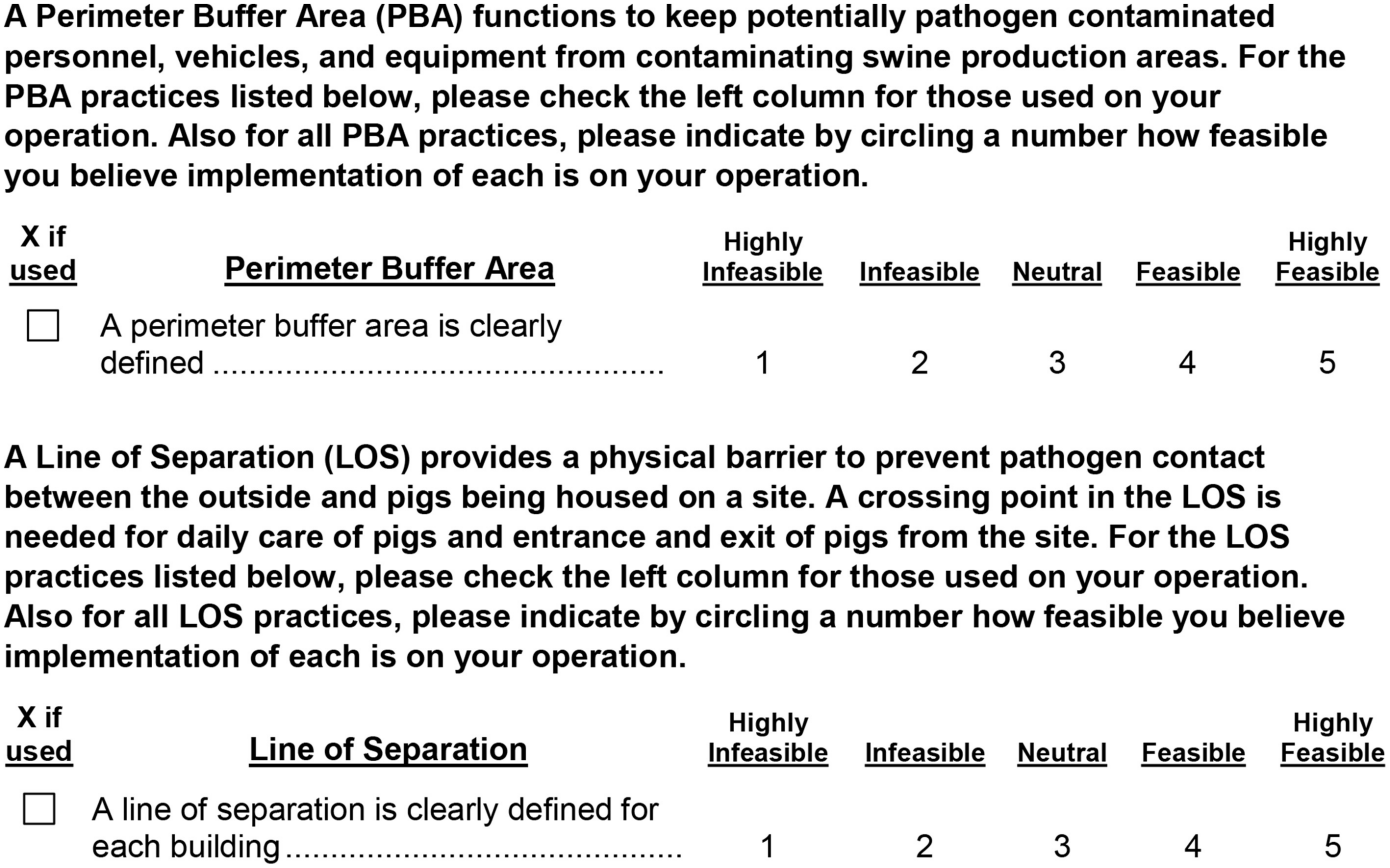
Example perimeter buffer area and line of separation questions from a 2017 survey of U.S. swine producers.

A self-assessment checklist for meeting PBA and LOS biosecurity performance standards was presented to each survey participant. Figure 1 provides examples of these survey questions, and the full list of PBA and LOS practices are displayed in Table 2. The survey contained Likert scale responses on a scale of 1 to 5 for feasibility of implementation, with 1 labeled as highly infeasible, 2 as infeasible, 3 as neutral, 4 as feasible, and 5 as highly feasible. These numerical responses were converted into categorical variables. Responses 1 and 2 were combined and converted into one infeasible label, 4 and 5 (feasible) were combined, and response 3 was not combined with other responses in order to more directly compare infeasible and feasible responses. Respondents who chose response 3, the neutral choice, might have been those who did not know or have definitive opinions about the feasibility of implementation (17). For questions regarding implementation on a producer's operation, responses were coded as binary variables equal to one if used on an operation and zero otherwise. Cross-tabulations were used to examine relationships between how feasible producers believe implementation is on their operation and if PBA and LOS practices are used on their operation.

**Table 2:** Cross-tabulations of PBA and LOS implementation and feasibility of implementation.

		**Mean**		**Implementation by feasibility rating**		
**Practice**	**Description**	***N***	**Implementation**	**Infeasible**	**Neutral**	**Feasible**
**PERIMETER BUFFER AREA (PBA)**						
PBADEFINED	A perimeter buffer area is clearly defined	317	0.391 (0.489)	0.161 (0.371)^a^	0.185 (0.391)^a^	0.569 (0.497)^b^
PBAENTRY	Access to perimeter buffer area is restricted through a single entry with a gate at the entrance which is locked when the facility is not attended	317	0.177 (0.382)	0.082 (0.276)^a^	0.117 (0.323)^a^	0.340 (0.476)^b^
PBAEQUIPMENT	All vehicles and equipment (not containing animals) entering the perimeter buffer area are documented to be clean, disinfected, and dried	315	0.229 (0.421)	0.066 (0.249)^a^	0.105 (0.309)^a^	0.479 (0.502)^b^
PBATRANSPORT	All animal transport vehicles are verified clean, disinfected, and dried before entry to the site	315	0.571 (0.496)	0.239 (0.430)^a^	0.233 (0.427)^a^	0.761 (0.427)^b^
**LINE OF SEPARATION (LOS)**						
LOSDEFINED	A line of separation is clearly defined for each building	309	0.605 (0.490)	0.327 (0.474)^a^	0.180 (0.388)^b^	0.778 (0.417)^c^
LOSLOCKED	Buildings are locked when no one is present	308	0.425 (0.495)	0.123 (0.331)^a^	0.064 (0.247)^a^	0.612 (0.488)^b^
LOSENTRY	One entry point has been established for personnel to cross the line of separation	308	0.688 (0.464)	0.235 (0.428)^a^	0.231 (0.427)^a^	0.876 (0.330)^b^
LOSANIMALS	All animals, including birds, are excluded from crossing the line of separation and contacting pigs	308	0.731 (0.444)	0.233 (0.427)^a^	0.409 (0.503)^b^	0.894 (0.309)^c^
LOSLOG	A visitor logbook is maintained by the site manager/owner	309	0.495 (0.501)	0.314 (0.471)^a^	0.125 (0.334)^b^	0.619 (0.487)^c^
LOSCLOTHING	Employees and visitors are instructed to change into site-specific coveralls or clothing and boots and wash hands when crossing to the pig side of the line of separation	309	0.770 (0.421)	0.361 (0.487)^a^	0.458 (0.509)^a^	0.859 (0.348)^b^
LOSCLOTHESPBA	When a site includes multiple pig buildings, site-specific clothing or coveralls and boots are put on within the perimeter buffer area and boots changed at each barn when crossing the line of separation	296	0.534 (0.500)	0.154 (0.364)^a^	0.190 (0.397)^a^	0.741 (0.439)^b^
LOSFOMITES	All equipment and other objects (including cell phones, jewelry, and electronics) that cross to the pig side of the line of separation are cleaned and disinfected, or come from a known clean source	303	0.419 (0.494)	0.137 (0.346)^a^	0.109 (0.315)^a^	0.663 (0.474)^b^
LOSCLEANING	Cleaning and disinfecting of animal rooms and buildings between groups of pigs is required	304	0.905 (0.294)	0.632 (0.496)^a^	0.313 (0.479)^b^	0.959 (0.198)^c^
LOSFEED	Feed is delivered and stored in bird, rodent, and insect proof containers/bins and feed spills are cleaned up	304	0.901 (0.299)	0.636 (0.492)^a^	0.467 (0.516)^a^	0.948 (0.223)^b^

Standard deviations are in parentheses.

a, b, c *Values within the same row with unique superscripts differ P < 0.10 according to Wilcoxon rank sum tests*.

### Binomial Logit Regression Analysis

Binomial logit regression analysis was used to determine the types of producers and operations most likely to adopt each of the following biosecurity practices: a written site-specific plan, PBA, and LOS. Two models were estimated to determine factors affecting development and implementation of a written site-specific plan, one for a plan for employees and one for a plan for delivery/service personnel. Separate models were estimated for each PBA practice (four total) and for each LOS practice (10 total), resulting in a binomial logit regression being estimated for each biosecurity practice. Descriptions and summary statistics for all 16 dependent variables (i.e., the aforementioned biosecurity practices) are provided in Table 2. Explanatory variables derived from the survey data are categorized as producer characteristics, operation characteristics, and risk attitudes. See Table 1 for descriptions and summary statistics of the explanatory variables.

Several of the explanatory variables require more explanation. Specifically, one novel contribution of this study is inclusion of an operation's past experience with any of the most common prevalent swine diseases. Of the five disease options included in the survey, porcine reproductive and respiratory syndrome (PRRS), and porcine epidemic diarrhea virus (PEDV) were the two diseases appearing on the U.S. National List of Reportable Animal Diseases for 2017 (18). Therefore, to control for past disease experiences, we include *REPORTABLE* as a binary explanatory variable that equals one if the producer had experienced a PRRS and/or PEDV outbreak on their operation in the last 3 years and zero otherwise.

Explaining producers' behavior in risky situations requires characterizations of risk attitudes. Exploratory factor analysis was conducted on responses to five Likert scale questions from the survey to assess swine producers' attitudes toward risk, especially in regards to how they manage their business financially. These questions included: (*a*) When marketing my hogs, I prefer financial certainty to financial uncertainty; (*b*) With respect to the conduct of my business, I prefer certainty to uncertainty; (*c*) I like “playing it safe”; (*d*) I am willing to take higher financial risks in order to realize higher average returns; and, (*e*) I like taking financial risks. The factor analysis (a principal factor analysis with a promax rotation) resulted in two factors that together explained more than 70% of the variation in the responses to the five Likert scale questions. The first factor was named *RISKAVERSE* due to high loadings on the first three questions, while the second factor was named *RISKACCEPTING* due to high loadings on the last two questions. In other words, the *RISKAVERSE* factor makes a meaningful contribution to the variation in responses to questions about risk aversion, while the *RISKACCEPTING* factor contributes meaningfully to the variation in responses to questions about risk acceptance. Scores for each factor, which are the sums of optimally weighted scores on the five questions (19), were estimated for each producer and included in each of the models as explanatory variables.

To account for expectations based on future disease events, a prospective risk attitude explanatory variable describes the number of times in the next 100 years that producers think a Tier 1 disease outbreak will occur in the U.S. swine industry. For this variable, responses were categorized into no outbreaks expected (*NOOUTBREAKS*), one outbreak expected (*ONEOUTBREAK*), and two or more outbreaks expected (*MUTIPLEOUTBREAKS*). This equates to a 0, 1, or 2% or more, respectively, perceived probability of a Tier 1 disease outbreak.

## Results and Discussion

### Feasibility of Biosecurity Implementation

Table 2 shows the percentage of producers responding to the survey that implemented each of the four PBA and ten LOS practices. Interestingly, PBA practice implementation was relatively low. Nearly 60% of producers verify all animal transport vehicles being clean, disinfected, and dried before entry to the site, but the remaining practices had mean adoption rates of less than 40%. Adoption of LOS practices was generally higher, however, with two practices having adoption rates above 90%.

Beliefs regarding how feasible implementation of each biosecurity practice is on his/her operation likely influences a producer's motivation to implement, as some measures could be perceived impractical or impossible (20–22). Relationships between feasibility ratings and biosecurity practice implementation were investigated using cross-tabulations (Table 2). The values in each of the cells in the table represent the percentage of those in the corresponding feasibility categorical group that have implemented the biosecurity practice. Not surprisingly, producers who believed implementation of a biosecurity practice was infeasible on their operation had lower implementation of that practice than producers who believed implementation was feasible.

For most practices, no statistically significant differences in implementation were detected between producers who believed implementation of a biosecurity practice was infeasible on their operation compared to those who were neutral about the feasibility of implementation. A few exceptions were found for LOS practices. For example, producers who were neutral about the feasibility of a line of separation being clearly defined for each building had a lower level of implementation of this practice than those who believed the practice was infeasible. Conversely, producers who were neutral about the feasibility of excluding all animals, including birds, from crossing the line of separation and contacting pigs had a higher level of implementation than those who believed the practice was infeasible. As previously indicated, a neutral response may indicate not knowing or having a definitive opinion about the feasibility of implementation, which may indicate a lack of knowledge of costs of implementation more generally. For example, fixed costs of biosecurity implementation are often relatively straight forward, but variable costs can be highly variable especially with respect to time (opportunity cost) and labor.

Comparing the relative implementation levels across feasibility ratings reveals several interesting patterns. Some producers are choosing not to implement certain biosecurity practices even though they consider the practices to be feasible to implement on their operation. On the other hand, some producers are choosing to implement biosecurity regardless of whether they deem the practice feasible to implement. This suggests that adoption of biosecurity measures on operations depends not only on feasibility of implementation, but on other motivations such as socio-economic factors.

### Determinants of Biosecurity Implementation

To examine the socio-economic factors related to adoption of SPS Plan biosecurity recommendations, we used binomial logit regressions. We focus on the marginal effects (Tables 3, 4) to show statistical significance of results and for interpretations of variables, where marginal effects were calculated as in Greene [(23), p. 734] for continuous variables and Greene [(23), p. 735] for binary variables. The marginal effects indicate the percentage change in probability either at the mean for continuous explanatory variables or for the “1” value for binary explanatory variables. Thus, one should interpret a positive effect as meaning that an increase in that explanatory variable has a positive impact on the probability of adopting the biosecurity practice in question.

**Table 3:** Adoption of written site-specific biosecurity plan and perimeter buffer area practices: marginal effects (SE).

**Variable**	**PLAN EMPLOYEES**	**PLAN PERSONNEL**	**PBA DEFINED**	**PBA ENTRY**	**PBA EQUIPMENT**	**PBA TRANSPORT**
AGE (Δ 1 year)	−0.001 (0.002)	−0.001 (0.002)	0.004^*^ (0.002)	−0.002 (0.002)	0.001 (0.002)	0.007^***^ (0.002)
COLLEGE	0.010 (0.053)	−0.038 (0.053)	0.027 (0.055)	0.014 (0.042)	−0.034 (0.051)	0.055 (0.058)
BREEDING (vs. FARROWFINISH)	0.064 (0.106)	0.085 (0.105)	−0.019 (0.107)	0.110 (0.097)	0.025 (0.098)	0.053 (0.120)
WEANFINISH (vs. FARROWFINISH)	0.046 (0.068)	−0.021 (0.076)	−0.163^**^ (0.075)	−0.030 (0.063)	−0.130^*^ (0.067)	−0.110 (0.077)
FINISH (vs. FARROWFINISH)	0.188^**^ (0.090)	0.190^*^ (0.106)	−0.040 (0.101)	−0.008 (0.081)	−0.033 (0.090)	−0.077 (0.111)
OTHEROPERATION (vs. FARROWFINISH)	−0.032 (0.136)	0.144 (0.139)	0.016 (0.139)	0.185 (0.130)	0.091 (0.139)	0.069 (0.150)
INTEGRATOR (vs. INDEPENDENT)	0.165 (0.105)	0.232^**^ (0.110)	0.253^**^ (0.118)	0.066 (0.084)	0.184 (0.123)	0.149 (0.118)
CONTRACTGROWER (vs. INDEPENDENT)	0.251^***^ (0.066)	0.232^***^ (0.068)	0.170^**^ (0.075)	0.091 (0.060)	0.067 (0.072)	0.044 (0.073)
OTHERBUSINESS (vs. INDEPENDENT)	0.202^**^ (0.103)	0.287^**^ (0.116)	0.151 (0.130)	0.066 (0.103)	0.210 (0.139)	0.247^**^ (0.117)
IOWA	−0.088 (0.063)	−0.038 (0.061)	−0.227^***^ (0.068)	−0.204^***^ (0.050)	−0.158^***^ (0.061)	−0.123^*^ (0.067)
ln(PRODUCTIONSITES)	0.065^**^ (0.027)	0.069^***^ (0.024)	−0.003 (0.026)	−0.013 (0.018)	−0.030 (0.024)	0.041 (0.028)
HIGHRATING	0.239^***^ (0.078)	0.321^***^ (0.062)	0.106 (0.075)	0.106^**^ (0.049)	0.165^***^ (0.055)	0.203^**^ (0.080)
REPORTABLE	0.002 (0.061)	−0.012 (0.061)	0.045 (0.062)	0.010 (0.045)	0.065 (0.054)	−0.018 (0.066)
RISKAVERSE	0.051^*^ (0.029)	0.034 (0.030)	0.058^*^ (0.032)	0.079^***^ (0.026)	−0.010 (0.028)	0.025 (0.032)
RISKACCEPTING	0.033 (0.028)	−0.046^*^ (0.028)	0.014 (0.030)	0.055^**^ (0.023)	0.019 (0.027)	0.059^*^ (0.031)
ONEOUTBREAK (vs. NOOUTBREAKS)	−0.115 (0.118)	−0.056 (0.103)	0.162 (0.118)	−0.048 (0.071)	0.107 (0.118)	−0.088 (0.120)
TWOOUTBREAKS (vs. NOOUTBREAKS)	−0.174^*^ (0.104)	−0.038 (0.101)	0.130 (0.093)	0.018 (0.070)	0.092 (0.088)	−0.077 (0.109)
*N*	263	262	263	262	262	262
Pseudo (McFadden's) *R*^2^	0.216	0.232	0.159	0.279	0.138	0.123
Predicted adoption rate	0.673	0.344	0.335	0.115	0.107	0.660
Actual adoption rate	0.605	0.385	0.395	0.176	0.240	0.580

*, **, *** *indicate statistical significance at p < 0.10, < 0.05, < 0.01, respectively*.

**Table 4:** Adoption of line of separation practices: marginal effects (SE).

**Variable**	**LOS DEFINED**	**LOS LOCKED**	**LOS ENTRY**	**LOS ANIMALS**	**LOS LOG**	**LOS CLOTHES**	**LOS CLOTHESPBA**	**LOS FOMITES**	**LOS CLEANING**	**LOS FEED**
AGE (Δ 1 year)	−0.004 (0.002)	−0.003 (0.002)	0.001 (0.002)	0.0004 (0.002)	0.00007 (0.002)	−0.001 (0.002)	−0.001 (0.003)	−0.001 (0.002)	0.001 (0.001)	0.001 (0.002)
COLLEGE	0.041 (0.055)	0.060 (0.057)	−0.031 (0.051)	0.010 (0.049)	−0.142^***^ (0.055)	−0.071 (0.046)	0.052 (0.060)	0.013 (0.057)	0.058 (0.036)	−0.078^**^ (0.039)
BREEDING (vs. FARROWFINISH)	0.197^**^ (0.090)	0.229^**^ (0.112)	0.270^***^ (0.057)	0.114 (0.079)	0.299^***^ (0.102)	0.148^**^ (0.075)	0.213^**^ (0.099)	0.251^**^ (0.118)	−0.057 (0.084)	0.053 (0.070)
WEANFINISH (vs. FARROWFINISH)	0.064 (0.071)	0.113 (0.076)	0.055 (0.065)	0.063 (0.060)	0.009 (0.073)	−0.030 (0.057)	0.109 (0.075)	−0.040 (0.077)	0.067^*^ (0.038)	0.120^**^ (0.048)
FINISH (vs. FARROWFINISH)^a^	0.098 (0.096)	0.091 (0.112)	0.011 (0.092)	0.120 (0.074)	0.153 (0.099)	0.079 (0.074)	0.220^**^ (0.095)	0.074 (0.111)	0.080^***^ (0.031)	
OTHEROPERATION (vs. FARROWFINISH)	0.141 (0.114)	0.293^**^ (0.127)	0.138 (0.090)	0.116 (0.083)	0.067 (0.138)	0.036 (0.097)	0.050 (0.149)	0.123 (0.145)	0.041 (0.051)	0.092^**^ (0.037)
INTEGRATOR (vs. INDEPENDENT)^b^	−0.031 (0.122)	0.119 (0.121)	−0.075 (0.123)	0.119 (0.087)	−0.124 (0.106)	0.028 (0.114)	−0.001 (0.124)	−0.055 (0.107)		
CONTRACTGROWER (vs. INDEPENDENT)	0.134^*^ (0.070)	0.192^***^ (0.071)	0.126^**^ (0.063)	0.134^**^ (0.061)	0.089 (0.071)	0.073 (0.055)	−0.074 (0.078)	0.071 (0.075)	0.095^***^ (0.036)	0.043 (0.059)
OTHERBUSINESS (vs. INDEPENDENT)^b^	0.181 (0.121)	0.364^***^ (0.120)	0.123 (0.119)	0.110 (0.102)	0.105 (0.140)	0.111 (0.100)	0.082 (0.147)	0.108 (0.142)		
IOWA	0.013 (0.063)	0.021 (0.063)	0.085 (0.058)	0.056 (0.058)	−0.151^**^ (0.064)	−0.045 (0.052)	0.097 (0.068)	−0.172^**^ (0.068)	−0.069^*^ (0.036)	−0.004 (0.048)
ln (PRODUCTIONSITES)	0.089^***^ (0.028)	0.078^***^ (0.027)	0.116^***^ (0.026)	0.086^***^ (0.027)	0.109^***^ (0.026)	0.064^**^ (0.026)	0.040 (0.029)	0.039 (0.026)	0.060^***^ (0.024)	0.007 (0.022)
HIGHRATING	0.264^***^ (0.080)	0.081 (0.077)	0.227^***^ (0.074)	0.224^***^ (0.077)	0.159^**^ (0.077)	0.182^***^ (0.068)	0.274^***^ (0.079)	0.211^***^ (0.074)	0.217^***^ (0.061)	0.236^***^ (0.076)
REPORTABLE	0.056 (0.065)	0.102 (0.066)	0.094 (0.060)	0.063 (0.058)	0.068 (0.063)	0.110^*^ (0.057)	−0.007 (0.070)	−0.001 (0.066)	0.028 (0.040)	0.117^**^ (0.058)
RISKAVERSE	0.070^**^ (0.030)	0.045 (0.032)	0.015 (0.028)	0.057^**^ (0.027)	0.057^*^ (0.031)	0.076^***^ (0.024)	0.059^*^ (0.033)	0.042 (0.032)	0.002 (0.019)	0.034 (0.023)
RISKACCEPTING	0.017 (0.030)	0.014 (0.030)	0.016 (0.027)	0.086^***^ (0.025)	−0.009 (0.029)	0.034 (0.024)	0.087^***^ (0.031)	0.051^*^ (0.031)	0.032^*^ (0.018)	0.055^**^ (0.024)
ONEOUTBREAK (vs. NOOUTBREAKS)	0.019 (0.110)	−0.081 (0.108)	−0.065 (0.122)	−0.009 (0.101)	−0.147 (0.103)	−0.040 (0.110)	0.00006 (0.122)	−0.108 (0.104)	0.039 (0.058)	0.064 (0.059)
TWOOUTBREAKS (vs. NOOUTBREAKS)	0.069 (0.104)	−0.062 (0.107)	−0.122 (0.096)	0.051 (0.097)	−0.172^*^ (0.102)	−0.053 (0.091)	−0.042 (0.113)	−0.103 (0.106)	−0.004 (0.057)	0.070 (0.085)
*N*	263	263	263	263	263	263	260	263	226	199
Pseudo (McFadden's) *R*^2^	0.166	0.143	0.202	0.205	0.215	0.242	0.097	0.141	0.432	0.320
Predicted adoption rate	0.719	0.304	0.791	0.859	0.479	0.878	0.604	0.342	0.951	0.950
Actual adoption rate	0.620	0.414	0.703	0.745	0.498	0.787	0.531	0.411	0.903	0.884

*, **, *** indicate statistical significance at p < 0.10, < 0.05, < 0.01, respectively.

a Dropped from the regression due to no variation for LOSFEED.

b Dropped from the regression due to no variation for LOSCLEANING and LOSFEED.

Although the marginal effects varied, several patterns emerged across the 16 estimated logit regressions. The signs on the marginal effects vary across biosecurity practices, but where the marginal effects were statistically significant, respondents were more likely to adopt certain practices as age increased. The effect of producer age on biosecurity adoption has provided mixed results in previous related research concerning adoption of other types of practices (24, 25). Older, more experienced swine producers may be expected to recognize the advantages of biosecurity practices, and thus to implement them. At the same time, those who have been in the business longer may be slower to adopt newer biosecurity practices.

Previous research has found that higher education results in greater adoption of technologies, management practices, and production systems (25, 26). However, we find that producers who hold a 4 year college degree were 14.2% less likely to maintain a visitor log book and 7.8% less likely to insure that feed is delivered and stored in bird, rodent, and insect proof containers/bins and feed spills are cleaned up. Although signs on the marginal effects for some other biosecurity practices suggest greater use by college-educated producers, these marginal effects are not statistically significant.

Operation type had a large influence on biosecurity adoption. When compared to farrow to finish operations, finishing operations were 18.8% more likely to always provide site-specific biosecurity procedures to employees and 19.0% more likely to always provide site-specific biosecurity procedures to delivery/service personnel. These results are somewhat unexpected given the health pyramid concept (27), which seeks to minimize the downstream effects of disease by controlling for disease toward the top of the pyramid and thus prioritizes the health of animals in the genetic nucleus and multiplication population, followed by farrowing and gestation, nursery, and lastly finishing animals. On the other hand, farrow to finish operations likely have fewer live animal inputs and retain some attributes of a closed herd which minimizes disease entry and introduction and could conceivably lessen the biosecurity needed. The results concerning breeding (i.e., breeding/farrowing or nursery) operations, however, reflect the health pyramid concept more closely, with breeding operations being more likely to adopt many LOS practices compared to farrow to finish operations. For example, breeding operations were 19.7% more likely to have a defined LOS than farrow to finish operations.

Business arrangement also had a large impact on adoption of biosecurity practices. For example, compared to independent producers, contract growers were more likely to always provide site-specific biosecurity procedures to employees (25.1%) and delivery/service personnel (23.2%). This makes sense considering that the U.S. swine industry is increasingly defined by contracts with growers to manage hogs provided and owned by a contractor. Production contracts typically spell out not only the length of a contract, terms for its renewal, and circumstances that would result in termination, but also specific provisions regarding which party is responsible for inputs like equipment, facilities, feeder pigs, feed, and other terms such as biosecurity policies. As such, the level of biosecurity might be institutionally fixed through production contract agreements.

More production sites as part of an operation was found to have a small but positive impact on biosecurity adoption suggesting the presence of economies of size. For instance, for every 10 additional production sites, a producer was about 0.7% more likely to always provide site-specific biosecurity procedures to employees and to delivery/service personnel and 0.9% more likely to have a defined LOS. This is consistent with Hennessy (28), Bottoms et al. (29), Nöremark et al. (30), and other studies that find larger operations are more likely to adopt biosecurity measures.

The Iowa variable, which represents geographical and pig density differences from other states, was generally found to decrease adoption rates ^4^. This seemingly contradicts the results of Bottoms et al. (29), who found that high herd density generally corresponded to higher biosecurity for sow herds in Ontario, Canada. Iowa is the most intensely populated hog production and pork processing state in the U.S. Geographical location of an operation and pig density in the area are two significant factors in the epidemiology of several diseases but, in general, producers do not have much control over them. In high pig density areas, it can be very difficult or impractical to maintain disease freedom from common endemic diseases. This is no excuse for lower biosecurity, but an appreciation of what is realistically achievable is essential and likely leads to mixed results when it comes to biosecurity implementation in high pig density regions.

Incidence of a reportable disease on an operation and high biosecurity self-rating were generally found to encourage biosecurity adoption. For example, compared to producers who did not experience PRRS and/or PEDV in the last 3 years, producers who had experienced at least one of these diseases were 11.0% more likely to instruct employees and visitors to change into site-specific coveralls or clothing and boots and wash hands when crossing to the pig side of the LOS and 11.7% more likely to have feed delivered and store in bird, rodent, and insect proof containers/bins and have feed spills cleaned up. This is consistent with statements made by Schulz and Tonsor (31) who suggest that the 2013-14 U.S. PEDV outbreak could have had the positive externality of encouraging biosecurity implementation. As expected, producers having rated their own biosecurity as high compared to their neighbors were more likely to adopt several of the recommended biosecurity measures. Of note, producers with a high biosecurity self-rating were 32.1% more likely to always provide site-specific biosecurity procedures to delivery/service personnel.

Risk attitudes were found to be significant determinants of biosecurity adoption. Results indicate that risk-averse producers, as determined via their responses to risk eliciting questions, were more likely to adopt the recommended biosecurity practices. Similarly, a risk-accepting attitude was positively correlated with adoption, the one exception being risk-accepting producers were 4.6% less likely to provide written site-specific biosecurity procedures for all delivery/service personnel. In general, these results suggest that regardless of how risky producers are in how they manage their business financially, they understand the importance of biosecurity measures needed on their operations and adopt accordingly.

Some producers may be more willing than others to adopt enhanced biosecurity practices because of the perceived risk of a high-consequence foreign animal diseases occurring. When compared to no expected outbreaks occurring, expecting one outbreak to occur did not statistically significantly impact biosecurity adoption. Producers who expected two or more outbreaks to occur, however, were less likely to adopt two of the recommended biosecurity practices. This latter result suggests a rather pessimistic view of high-consequence foreign animal diseases occurring in the United States could be a deterrent to biosecurity adoption for some producers and for some particular biosecurity practices.

As Mankad (32) highlights, there is a need to incorporate psychological, social and cognitive factors on decision making related to biosecurity and management practices. Our results emphasize the important role of risk attitudes and perceptions in explaining biosecurity adoption behavior which is valuable since studies of the factors leading to the adoption of technology and management practices by producers all too often focus only on the explanatory role of typical producer demographics.

The last two rows of Tables 3, 4 report “predicted adoption rate,” and “actual adoption rate,” which are useful in evaluating the capability of these models to predict biosecurity adoption. The “predicted adoption rate” is the proportion of producers that each binomial logit regression predicted would adopt a particular biosecurity practice, while “actual adoption rate” is the proportion of producers who adopted that practice as recorded in the survey responses. As these statistics demonstrate, the models reasonably predicted the adoption rate in most cases. In particular, 60.5% of producers always provide site-specific biosecurity procedures to employees, and the model predicted 67.3% of producers would adopt this practice. Results such as these lend credence to the logit procedure used in this analysis. That said, not all of the 16 models performed this well. Additionally, some variables are statistically significant in some models but not in others when we might expect the impact of a variable on adoption to be similar for some (or even most) practices. The results from the logit regressions (and the previously discussed feasibility analysis) indicate that socio-economic factors (producer and operation demographics and producer attitudes and perceptions) play a significant role in biosecurity adoption, but still other factors could be drivers of adoption.

### Complementarity of Biosecurity Practices

Thus far, adoption of biosecurity practices have been considered independently. The complementary nature of adoption of the enhanced biosecurity practices could be evaluated to reveal how adoption of one practice influences adoption of another practice. An analysis of complementarity was completed utilizing conditional probabilities (33, 34), with complementarity of adoption being demonstrated by following Chihara and Hesterberg (35) to show statistically significant differences between adoption rates of one practice for adopters and non-adopters of a second practice. Results are reported in Tables 5–7.

**Table 5:** Adoption rates of providing a written site-specific biosecurity plan, a defined perimeter buffer area, and a defined line of separation (in italics) given non-adoption or adoption of the other practices (in bold).

	**Non-Adoption (%)**	**Adoption (%)**
PLANPROVIDED^*^		
*PBADEFINED*	26.5	63.3
*LOSDEFINED*	52.0	75.0
**PBADEFINED**		
PLANPROVIDED^*^	22.7	58.5
*LOSDEFINED*	49.2	77.6
**LOSDEFINED**		
PLANPROVIDED^*^	23.2	45.5
*PBADEFINED*	22.2	50.5

* Adoption of PLANPROVIDED means that a producer always provides site-specific procedures to all employees and to all deliver/service personnel. Of the 337 respondents, 37.09% adopted PLANPROVIDED.

*All differences (rate given adoption – rate given non-adoption) statistically significant at p ≤ 0.01*.

Consider the following two LOS practices—clearly defining a LOS for each building (*LOSDEFINED*), and having one established entry point for personnel to cross the line of separation (*LOSENTRY*). Intuitively, having a clearly defined LOS is basically required for having one entry point for crossing, so one could reasonably expect that adoption of one of these practices impacts the adoption of the other. The data corroborates this prediction. As shown in Table 7, of the subset of producers who reported having a defined LOS, 91.1% reported also having one entry point for crossing. Compare this to the subset of producers who reported not having a defined LOS, of which only 33.6% reported having one entry point for crossing. As expected, adoption of these two biosecurity practices goes hand in hand, and this result is not unique. Consider two other practices for which adoption could be expected to be related—vehicles and equipment entering the PBA are verified to be clean, disinfected, and dried before entry to the site (*PBAEQUIPMENT*), and transport vehicles entering the PBA are verified to be clean, disinfected, and dried before entry to the site (*PBATRANSPORT*). As Table 6 shows, the conditional probability of verifying that vehicles and equipment entering the PBA are clean, disinfected, and dried given that all transport vehicles entering the PBA are verified to be clean, disinfected, and dried is six times higher (36.8 vs. 5.8%) than the conditional probability of verifying that vehicles and equipment entering the PBA are clean, disinfected, and dried given that the practice of verifying that all transport vehicles entering the PBA are clean, disinfected and dried has not been adopted. Once again, adoption of one of these practices clearly impacts the adoption of the other.

**Table 6:** Adoption rates of perimeter buffer area practices (in italics) given non-adoption or adoption of the other perimeter buffer area practices (in bold).

	**Non-Adoption (%)**	**Adoption (%)**
**PBADEFINED**		
*PBAENTRY*	7.7	32.3
*PBAEQUIPMENT*	11.3	41.5
*PBATRANSPORT*	43.6	78.3
**PBAENTRY**		
*PBADEFINED*	32.8	73.7
*PBAEQUIPMENT*	16.8	55.4
*PBATRANSPORT*	53.0	78.6
**PBAEQUIPMENT**		
*PBADEFINED*	30.6	71.1
*PBAENTRY*	10.1	40.8
*PBATRANSPORT*	47.4	89.5
**PBATRANSPORT**		
*PBADEFINED*	20.3	54.3
*PBAENTRY*	8.7	23.7
*PBAEQUIPMENT*	5.8	36.8

*All differences (rate given adoption – rate given non-adoption) statistically significant at p ≤ 0.01*.

**Table 7:** Adoption rates of line of separation practices (in italics) given non-adoption or adoption of the other line of separation practices (in bold).

	**Non-adoption (%)**	**Adoption (%)**
**LOSCLEANING**		
*LOSDEFINED*	17.2	65.0
*LOSLOCKED*	10.3	45.9
*LOSENTRY*	20.7	73.4
*LOSANIMALS*	17.2	79.1
*LOSLOG*	10.3	53.4
*LOSCLOTHING*	24.1	82.7
*LOSCLOTHINGPBA*	14.3	56.9
*LOSFOMITES*	10.3	44.7
*LOSFEED*	51.7	94.0
**LOSDEFINED**		
*LOSLOCKED*	18.3	57.8
*LOSENTRY*	33.6	91.1
*LOSANIMALS*	48.8	89.1
*LOSLOG*	27.0	63.5
*LOSCLOTHING*	53.2	92.2
*LOSCLOTHINGPBA*	28.9	68.9
*LOSFOMITES*	12.3	60.3
*LOSCLEANING*	80.5	97.4
*LOSFEED*	79.7	96.8
**LOSENTRY**		
*LOSDEFINED*	17.0	80.6
*LOSLOCKED*	13.0	55.0
*LOSANIMALS*	36.4	89.9
*LOSLOG*	25.0	60.4
*LOSCLOTHING*	49.0	89.4
*LOSCLOTHINGPBA*	25.0	65.9
*LOSFOMITES*	13.3	54.5
*LOSCLEANING*	76.5	97.2
*LOSFEED*	74.5	97.2
**LOSANIMALS**		
*LOSDEFINED*	24.7	73.7
*LOSLOCKED*	16.5	51.7
*LOSENTRY*	25.9	84.4
*LOSLOG*	22.4	58.6
*LOSCLOTHING*	42.4	89.2
*LOSCLOTHINGPBA*	20.3	64.7
*LOSFOMITES*	15.7	50.7
*LOSCLEANING*	71.1	97.8
*LOSFEED*	69.9	97.4
**LOSCLOTHING**		
*LOSDEFINED*	20.3	72.5
*LOSLOCKED*	13.5	50.8
*LOSENTRY*	31.1	79.8
*LOSANIMALS*	33.8	85.2
*LOSLOG*	8.1	61.5
*LOSCLOTHINGPBA*	23.2	61.7
*LOSFOMITES*	11.3	50.4
*LOSCLEANING*	69.0	97.1
*LOSFEED*	73.2	95.0
**LOSFEED**		
*LOSDEFINED*	19.4	65.1
*LOSLOCKED*	12.9	45.9
*LOSENTRY*	19.4	73.9
*LOSANIMALS*	19.4	79.3
*LOSLOG*	9.7	53.7
*LOSCLOTHING*	38.7	81.5
*LOSCLOTHINGPBA*	16.1	57.1
*LOSFOMITES*	12.9	44.6
*LOSCLEANING*	54.8	94.7
**LOSFOMITES**		
*LOSDEFINED*	41.2	88.4
*LOSLOCKED*	30.2	59.7
*LOSENTRY*	53.3	89.9
*LOSANIMALS*	61.5	89.8
*LOSLOG*	29.7	77.5
*LOSCLOTHING*	65.4	93.8
*LOSCLOTHINGPBA*	37.4	75.2
*LOSCLEANING*	85.7	97.7
*LOSFEED*	85.2	96.9
**LOSCLOTHINGPBA**		
*LOSDEFINED*	39.9	78.3
*LOSLOCKED*	32.2	51.6
*LOSENTRY*	49.7	85.1
*LOSANIMALS*	55.6	90.1
*LOSLOG*	35.0	62.7
*LOSCLOTHING*	62.9	90.1
*LOSFOMITES*	21.7	58.4
*LOSCLEANING*	83.2	97.5
*LOSFEED*	81.8	96.9
**LOSLOG**		
*LOSDEFINED*	43.2	78.2
*LOSLOCKED*	30.9	53.8
*LOSENTRY*	53.4	84.0
*LOSANIMALS*	59.3	87.7
*LOSCLOTHING*	58.0	96.2
*LOSCLOTHINGPBA*	39.2	66.9
*LOSFOMITES*	18.5	64.9
*LOSCLEANING*	83.5	98.1
*LOSFEED*	82.3	98.1
**LOSLOCKED**		
*LOSDEFINED*	44.0	82.8
*LOSENTRY*	53.0	90.2
*LOSANIMALS*	61.2	89.6
*LOSLOG*	39.1	62.7
*LOSCLOTHING*	65.2	92.5
*LOSCLOTHINGPBA*	44.6	64.3
*LOSFOMITES*	29.1	58.3
*LOSCLEANING*	85.5	97.7
*LOSFEED*	84.9	97.0

*All differences (rate given adoption – rate given non-adoption) statistically significant at p ≤ 0.01*.

High degrees of complementarity are more or less universal across all practices, even for practices that are not as obviously related as the pairs of practices detailed in the previous paragraph. Consider this time the practices of always providing site-specific biosecurity procedures to both employees and delivery/service personnel (*PLANPROVIDED*) and of clearly defining a PBA (*PBADEFINED*). Even though there is no evident relationship between the two practices, the subset of producers who always provide site-specific biosecurity procedures has a 63.3% adoption rate for clearly defining a PBA, while the subset of producers who do not always provide site-specific biosecurity procedures has a much lower adoption rate of 26.5% for defining a PBA (Table 5).

It is possible that complementarity occurs because the use of one practice increases the marginal efficacy of another practice. However, as Pruitt et al. (33) indicate, it could also be because some people are simply “adopters” of practices. In either case, the complementarity results show that adoption of specific biosecurity practices often go hand in hand.

## Conclusion

Given that biosecurity adoption in the past in the United States has largely been precautionary and voluntary, this study provides key insights into the very complex and ever-changing issue of biosecurity adoption. Producer attitudes about feasibility of implementation, producer and operation demographics, risk attitudes and perceptions, and complementarity of practices were all shown to play a meaningful role in whether or not a producer adopted recommended SPS Plan biosecurity practices. As was demonstrated, not one of these factors provides the entire picture by itself and there are many factors at play when producers weigh biosecurity adoption. Further complicating the situation is that biosecurity adoption is not static. In fact, producer attitudes and adoption rates may have meaningfully changed since the data for this study was collected, especially in response to the recent outbreaks of ASF in China.

Keeping these complexities in mind is of utmost importance, especially at a time when, for better or for worse, the precautionary and voluntary biosecurity paradigm in the United States appears to be shifting. For example, governing bodies in the United States have not historically mandated (either directly or indirectly) biosecurity adoption in response to concerns with antimicrobial resistance the same way their European counterparts have [see European Commission, (36)]. In recent years, however, policies such as the Veterinary Feed Directive—which has been shown by Schulz and Rademacher (37) to cause producers to modify biosecurity—are being considered and implemented by U.S. governing bodies. As another example, during the 2014–2015 Highly Pathogenic Avian Influenza outbreak in the U.S., indemnity payments to producers were made conditionally on producers providing evidence that they were using biosecurity meant to prevent the spread of the disease when it was discovered in their flocks (38). Given the complicated nature of this issue, this study could provide educators and policy makers with vital information as they think about such policies going forward.

The complexity of this issue also means there are plenty of opportunities for further research. In our survey, producers indicated if they used the recommended biosecurity practices on their operation. Future surveys should consider framing of the question with respect to peacetime (i.e., a normal operating environment) and during an animal health emergency (i.e., acute animal health crisis actively impacting interstate and international trade). That is, instead of presenting binary options “being used” or “not being used,” perhaps producers could be asked to select from “being used,” “not currently being used but could be if needed,” or “not feasible to implement.” This approach would help, in part, determine producers' evaluations of industry-wide costs of not adopting biosecurity. Livestock operations obviously have a strong interest in remaining disease-free for a variety of reasons, but operations may not take into account how their actions affect other operations. If a producer is shown to always use a practice, it may be that the producer has a better understanding (or higher valuation) of industry-wide costs of a disease outbreak compared to a producer who only implements biosecurity during an outbreak. Knowing how many producers undervalue industry-wide costs of an outbreak would be very helpful to program administrators and educators.

Future work should also consider collecting information on farm-level costs of biosecurity implementation as this may help to better explain adoption than, more generally, self-assessments of the perceived feasibility of adoption on an operation. For example, biosecurity investments entail a mixture of fixed and variable costs. By knowing farm-level fixed and variable cost estimates, economic tradeoffs could be considered and the relative influence of each for biosecurity adoption identified. Furthermore, knowing costs would help inform cost-sharing schemes related to animal disease mitigation efforts, where biosecurity is intended to be a factor in the cost-sharing strategies.

The logit and complementarity analyses conducted here, although robust, have limitations. In particular, while the complementarity analysis definitely demonstrates that adoption of practices go together, one has to be careful when drawing conclusions about causality when only considering conditional probabilities. Multinomial or multivariate logit models could be used instead of binomial logit models and conditional probabilities, but as Pruitt et al. (33) also found, such analysis is made difficult by the sheer number (in this case, 16) of recommended practices. Also of note is that this analysis is agnostic about the relative importance of different practices. For example, clearly defining a LOS for each building may or may not be more important for overall biosecurity than maintaining a visitor logbook, but the analysis in this study makes no value judgment. Making an assumption about the relative importance of practices could allow for further analysis to proceed. By weighting each practice the same for overall operation biosecurity, one could conduct a count data regression analysis as in Gale (39) to look for jointness in adoption. Alternatively, a weighting system could be applied such as in Postma et al. (14) to give an overall biosecurity rating to each producer before performing subsequent analysis. Future research avenues such as these could enhance and extend the results in this study, providing additional valuable insights about biosecurity adoption.

Future producer and industry leader education efforts may be more targeted by incorporating findings from this study. These refined efforts may lead to a clearer understanding by producers of biosecurity's many decisions and ultimately improved decision-making regarding biosecurity adoption and compliance efforts. Similarly, future efforts to develop new technologies, programs, or protocols to enhance individual firm and industry-wide biosecurity should heed these results. Development or marketing of biosecurity options that producers are unlikely to widely implement is a missed opportunity, while alternatively focusing efforts on options more likely to be utilized is an improved possibility if the results from this study are properly leveraged.

## Ethics Statement

This study was carried out in accordance with the recommendations of The Belmont Report: Ethical Principles and Guidelines for the Protection of Human Subjects of Research or other appropriate ethical standards recognized by federal departments and agencies that have adopted the Federal Policy for the Protection of Human Subjects. The protocol was approved by the Iowa State University Institutional Review Board. All methods for data collection and gathering were performed in accordance with the relevant regulations and in compliance with the received guidelines.

## Author Contributions

All authors listed have made a substantial, direct and intellectual contribution to the work, and approved it for publication.

### Conflict of Interest Statement

The authors declare that the research was conducted in the absence of any commercial or financial relationships that could be construed as a potential conflict of interest.

## Footnotes

^1^For more information refer to the SPS Plan website at http://www.securepork.org/.

^2^These three concepts were outlined in “SPS Plan Update 2013, DRAFT SPS Plan: Appendix A Producer Biosecurity.” The 2013 draft expounds on these recommendations, highlighting the importance of a PBA and a LOS (particularly) and listing various components of each. As such, the PBA and LOS are the focus of the feasibility analysis. Since 2013, the SPS Plan has gone through various updates, with the current iteration of the SPS Plan highlighting *four* biosecurity recommendations: the previously-mentioned three items, as well as the explicit recommendation for a designated Biosecurity Manager (4).

^3^The SPS Plan defines a perimeter buffer area (PBA) as an outer control boundary around swine buildings designed to restrict disease transmission near such buildings and a line of separation (LOS) as a control boundary to restrict disease transmission into areas where hogs can be exposed (4). In practice, a PBA is often a fence or other physical barrier with defined access points around swine buildings while a LOS is often the walls of the buildings themselves (4). See Secure Pork Supply [(4), pg. 5] for an illustrative diagram of a PBA and a LOS.

^4^A custom link survey was utilized in Iowa. In an effort to increase survey response, expand distribution to include swine producers from other states, and respective state associations' reticence concerning access to members an open link survey was utilized for swine producers outside of Iowa. As such, we were only able to identify Iowa operations with certainty with producers outside of Iowa not reporting which states(s) their production sites are located in or reporting multiple states. While the Iowa variable does allow us to roughly identify how the use of biosecurity measures differ across geographic location, future research should further explore geographical differences with the inclusion of additional states or regions.
